# P06 The 15-valent pneumococcal conjugate vaccine (PCV) V114 induces cross-reactive antibodies against pneumococcal serotype 6C

**DOI:** 10.1093/jacamr/dlac133.010

**Published:** 2023-01-19

**Authors:** Yaru Shi, Katrina Nolan, Robert L Burton, Rocio Murphy, Natalie Banniettis, Luwy Musey, Ulrike Buchwald

**Affiliations:** Merck & Co. Inc., Kenilworth, NJ, USA; Merck & Co. Inc., Kenilworth, NJ, USA; SunFire Biotechnologies LLC, Birmingham, AL, USA; Merck & Co. Inc., Kenilworth, NJ, USA; Merck & Co. Inc., Kenilworth, NJ, USA; Merck & Co. Inc., Kenilworth, NJ, USA; Merck & Co. Inc., Kenilworth, NJ, USA

## Abstract

**Background:**

Pneumococcal serotype 6C has structural similarity with serotype 6A and, to a lesser degree, serotype 6B. The 13-valent PCV (PCV13), which contains serotypes 6A and 6B, induces cross-reactive opsonophagocytic killing activity (OPA) antibodies to serotype 6C, with an observed decline in 6C disease incidence. V114 is a 15-valent PCV containing the 13 serotypes in PCV13 and two unique serotypes (22F and 33F). This study evaluated whether V114 elicits cross-reactive OPA antibodies to serotype 6C.

**Methods:**

OPA antibodies to serotypes 6A, 6B, and 6C were evaluated in serum samples taken pre-vaccination and 30 days post-vaccination (single dose) from adults ≥50 years of age, and 30 days pre- and post-dose 4 from toddlers 12–15 months of age at fourth dose, who received V114 or PCV13 in the adult and paediatric V114 phase II proof-of-concept studies, respectively. The degree of inhibition of OPA antibody activity for serotypes 6A, 6B, and 6C after adsorption with serogroup 6 polysaccharides was evaluated in adult samples.

**Results:**

Samples from 250 adults (V114, *n*=150; PCV13, *n*=100) and 150 paediatric participants (V114, *n*=100; PCV13, *n*=50) were included. In both the adult and paediatric populations, observed OPA geometric mean titres (GMTs) to serotypes 6A, 6B, and 6C were comparable across vaccination groups; however, the cross-reactive response to serotype 6C was stronger in infants than adults. The inhibition analysis demonstrated the specificity of antibody responses.

**Conclusions:**

V114 induces cross-reactive antibodies to serotype 6C in adult and paediatric populations that are specific and comparable to those induced by PCV13.

